# Ko te mana o te tamaiti te aro o tātou mahi: Listening to voices from Tai Tokerau to re-frame literacies

**DOI:** 10.1007/s40841-023-00290-7

**Published:** 2023-06-06

**Authors:** Maia Hetaraka, Selena Meiklejohn-Whiu, Melinda Webber, Rebecca Jesson

**Affiliations:** 1 Te Puna Wānanga, Waipapa Taumata Rau, University of Auckland, Auckland, New Zealand; 2 School of Curriculum and Pedagogy, Faculty of Education and Social Work, Waipapa Taumata Rau, University of Auckland, Auckland, New Zealand; 3 Tai Tokerau Campus, Waipapa Taumata Rau, University of Auckland, Whangarei, New Zealand

**Keywords:** Literacies, Mana, Te Ao Māori

## Abstract

Western literacy theories and models often reflect Eurocentric notions of literacy and literacy practices. In Aotearoa New Zealand, the prevalence of these conceptualisations is linked to issues of power and result in a narrow and inaccurate framing of Māori tamariki (children). In this article Tiritiria, a Māori philosophical view of knowledge, knowledge generation and knowledge exchange is used alongside Webber and Macfarlane’s (2020) Mana Model to challenge this dominant framing of literacy. Using the whakataukī ‘Ko te mana o te tamaiti te aro o tātou mahi', translated literally as ‘Let the mana of the child guide our work’, tamariki Māori are (re)positioned as maurea (treasures) to further support the (re)framing of literacies. In this study we focus on listening to the voices of whānau Māori from Te Tai Tokerau (Northland, New Zealand), including the voices of tūpuna (ancestors). Through a developing understanding of tiritiria and an analysis of data sets from Tai Tokerau a nascent definition of literacies, as multitudinous, practical enactments of tirititia, emerged. Findings indicated that Māori literacy practices (both traditional and contemporary) move beyond subject learning, to incorporate multiple interpersonal, cultural, environmental and textual processes of knowledge transfer which affirm the inherent and inherited mana of tamariki.

## Introduction

The present study seeks to understand perspectives of childhood literacies as expressed by Tai Tokerau kaumātua (elders), whānau (extended family), tamariki (children), kaiako (teachers), and tūpuna (ancestors). The authors are part of the international Northern Oral Language and Writing through Play (NOW Play) project consortium (https://now-play.org/) which originated in Canada and includes indigenous communities from Canada, Sweden, and Aotearoa New Zealand. The team authoring this paper form one Aotearoa branch of the project (Hetaraka et al., 2023). The focus of our research is on literacy for tamariki Māori in Aotearoa, and the development of those literacies within the beginning years of interaction with the schooling system. Battiste (2005) asserts the value of utilising dynamic indigenous knowledge frameworks to create “…a new, balanced centre and a fresh vantage point…” (p. 3). Hare (2012) building on this position, suggests that an indigenous knowledge framework can highlight literacy experiences of indigenous communities. To arrive at an understanding on the early years of literacy learning for Māori children, a decolonised te ao Māori (Māori world) perspective of knowledge and literacy must first be examined.

Māori is a term used to describe the peoples indigenous of Aotearoa. The term acknowledges interconnected whakapapa (genealogy) as well as a shared politicised identity as indigenous and colonised peoples. In this paper, while the term Māori is used in a general way, we acknowledge the many independent yet connected nations, tribes and people that are ‘Māori’. All hapū (small tribal groups) and iwi (large tribal groups) have their own histories, knowledge bases and wisdom, so the use of the term ‘Māori’ throughout does not imply homogeneity but instead acknowledges that shared experiences enable some general conclusions to be drawn. We acknowledge the unique knowledge of the people of Tai Tokerau from whom the development of our understandings in this context have derived.

As researchers of literacy learning for tamariki Māori, key assumptions underpin all the meanings that interanimate in the research space; assumptions about (i) children, (ii) learning and (iii) literacy. Each of these assumptions underpin particular positionings that express worldviews: views of children and childhood, views of knowledge and how we learn it, and views of literacy and what it encompasses. It was our contention that each of these aspects of the research focus needed examination, for what they mean from a Māori worldview. The interrogation of te ao Māori assumptions is essential in order to challenge and progress the incorrect notion that literacy learning (like mathematics education) is simply a curriculum area, value-free and inconspicuous.

We intentionally (re)positioned Māori children so that we could think about them and their gifts from a te ao Māori perspective of potentiality, –that is, seeing children’s timeless selves, as inherently and inherited-ly literate (Hetaraka et al., 2023). This conception positions children as maurea (unique treasures), which stems from pre-colonial teachings, that children were to be held in high regard (Papakura, 1986). Children represent the future of iwi, and their growth impacts on the collective. Iwi and hapū have always given attention and priority to the health, strength, knowledge, and mana of children (Pihama & Lee-Morgan, 2022). Thus, in order to understand literacies in Aotearoa, we begin with a foundational premise: that Māori children must once again be seen as maurea, –treasures equipped with unique skills, abilities and knowledge who must be protected and nurtured for the prosperity of the collective.

To frame our theory of learning we draw upon the notion of *Tiritiria* as the theory of both knowledge and knowledge transfer (Hetaraka et al., 2023). From a Māori perspective, knowledge is seen as diverse and immeasurable, encompassing multiple realms. From the perspective of tiritiria, having only one way of knowing is both limited and limiting. Knowledge diversity is a concept often rejected by a dominant western philosophy, which has a history of defining legitimate knowledge as western knowledge (Akena, 2012). Instead we argue that reframing thinking around what knowledge is, and whose knowledge is being noticed and affirmed, is vital for our education settings. Therefore, our second premise for the paper is that knowledge is diverse and understood in multiple ways.

With tiritiria as the knowledge and knowledge transfer theory, we next need to reframe a definition of literacy and consider how notions of literacy embrace the multiplicity of ways that knowledges can be expressed, shared and learned, by and with children who are inherently and inherited-ly literate. This reframing emphasises the importance of seeing literacy as a carrier of knowledge, and as a way of accessing knowledge. Thinking of literacy in this way illuminates how unexamined definitions of literacy operate as a gate keeper, defining both the value of knowledge, and its means of expression (the literacy). Tiritiria re-positions tamariki as both the origin and outcome of theory, the consumer and producer of knowledge, and as both teacher and learner of past, present and future literacies. Tiritiria contends that tamariki as 'maurea' both inherit the knowledge systems of their tūpuna, and inherently create new systems of knowledge in relation and response to the world around them.

## Inherently and Inherited-ly Literate: Opportunities to Manifest and Express Mana

To operationalise the concept of inherently and inherited-ly literate, the team drew from Webber and Macfarlane’s (2020) Mana Model, which expresses five key conditions for thriving. The concept of mana lies at the heart of Māori self-worth and wellbeing. Royal (2006) has described it as a “quality, energy or consciousness in the world which can be harnessed and expressed in human activities through acts of generosity and wisdom” (p. 8). Similarly, Dell (2017) defined mana as “a person’s influence and profound ability to impact upon, affect, and positively transform the lives of others” (p. 93).

**Fig. 1:**
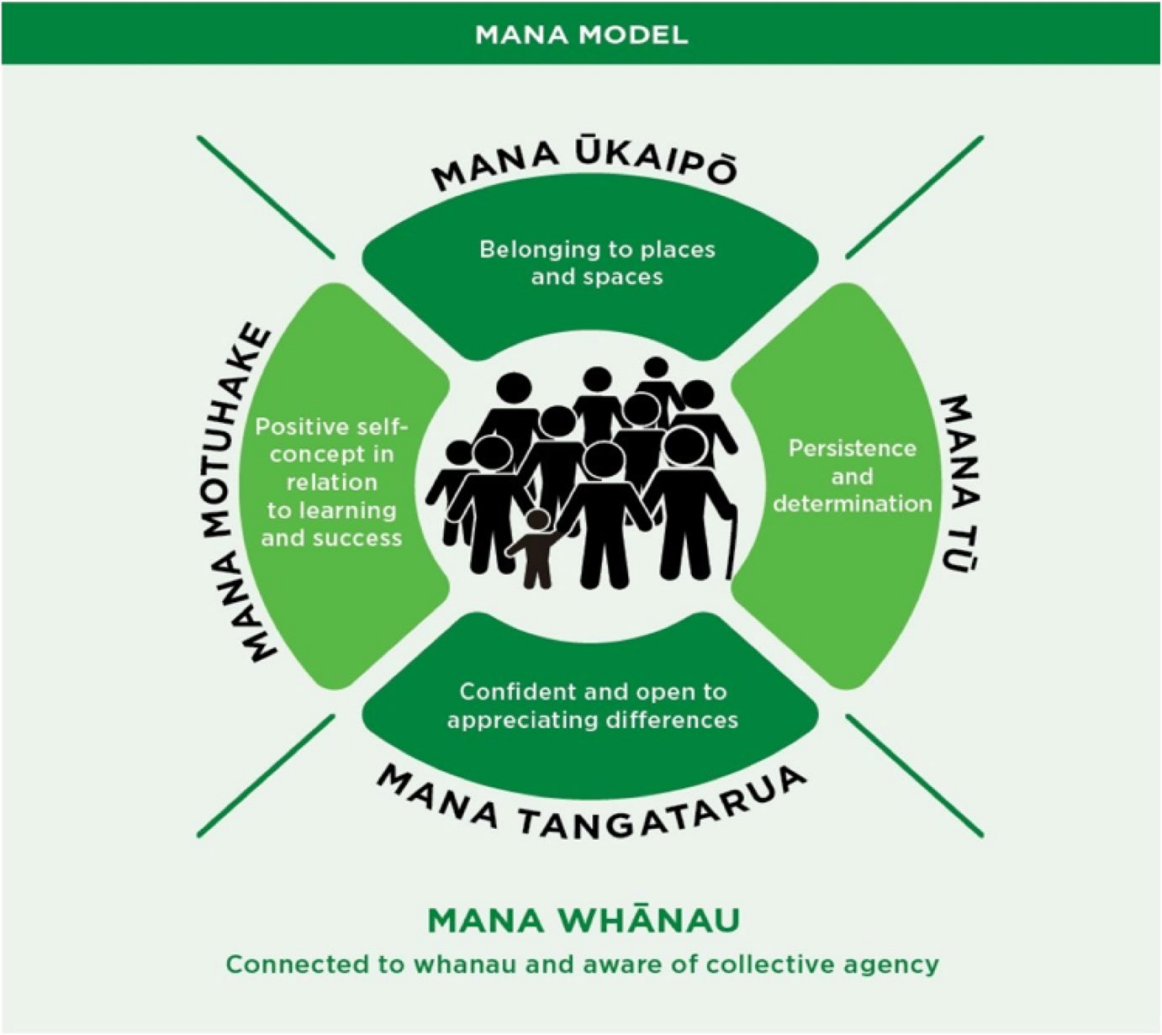
The Mana Model (Webber and Macfarlane, 2020)

The Mana Model is a kaupapa Māori informed social-psychological model, identifying “five key components concerning the optimal personal, familial, school, and community conditions” for Māori thriving (Webber & Macfarlane, 2020 p. 26). The model proposes that Māori children’s thinking, behaviour and wellbeing are underpinned by their sense of mana–self-efficacy, purpose, pride and belonging. The model (see Fig. 1) is strengths-based and derived from a te ao Māori worldview and in this research, focuses on operationalising the theory of children’s psycho-social development through understanding collective conditions to thrive.

The key components in the Mana Model are Mana Whānau (relationship and connection with others), Mana Motuhake (positive self-concept and a sense of embedded achievement), Mana Tū (tenacity and self-esteem), Mana Ūkaipō (belonging and connectedness to place), and Mana Tangatarua (diverse knowledge and skills) (Webber & Macfarlane, 2020, p. 26). Conceptually, each of these aspects of mana both contribute to, and interact with, children’s developing literacy, conceived here as the vehicle for knowledge building and sharing, in each of the realms expressed as components of the model.

The conditions for thriving as outlined in the Mana Model are aspirational, and we acknowledge that for many Māori having their mana recognised and esteemed has been disrupted. However, in our examination of these data we heard multiple kaumātua, whānau, students and teachers identify each of the realms of the Mana Model as being important to the academic, social and spiritual growth of individuals and the collective.

## Research Processes

Underpinned by the Māori worldview embedded in tiritira and the Mana Model, our research questions sought to interrogate both the nature of literacies from a Māori perspective, as well as the experiences that enable literacy development in informal settings (for the purposes of this project informal teaching and learning is referred to as ‘play’). Our key research questions were:

- How do whānau, notice, acknowledge, and affirm the mana of tamariki Māori through literacy learning experiences?
- What experiences facilitate ‘play’ that builds literacy learning?

We began the process by considering existing expressions of Māori literacies knowledge already accessible to us. Three Tai Tokerau based studies (Hetaraka, 2008, 2020; Webber et al., 2021) were utilised as a starting point in our search. These three existing data sets contained rich narratives that had been collected across previous studies, representing the voices of tūpuna, kaumātua, whānau, kaiako and tamariki (children). Although tamariki voices are not quoted explicitly in this paper, we contend that they are always there. Māori worldview positions tamariki as central to te pā korari (a flax plant). This metaphor for whānau flourishing positions tamariki (te rito o te korari) as the centre shoot, always at the heart of decision making and activities. In this way, tamariki voice is omnipresent, importantly and always at the centre of the thoughts shared by these participants. These data were brought together as the foundations for developing the NOW Play Aotearoa model of literacies for tamariki Māori.

From the beginning, kaupapa Māori and te ao Māori concepts have informed the thematic analysis of these data sets. One of the tikanga employed early in this project was the importance of āta hakarongo-to carefully listen to all the voices we had access to. Waves of thematic analysis of the narratives and survey data informed our developing framework. In the first wave, the Mana Model (Webber & Macfarlane, 2020) was used as an overarching analysis tool to enable us to listen for narratives that spoke to potentials for literacies. Within each narrative we were able to consider both the nature of the literacies that were presented, and the opportunities that children were deliberately provided with to engage and express these literacies. Each of the components of the Mana Model were used as a lens through which we could question the transcripts and identify themes that reflected the interconnected aspects of the model. This first wave formed the data identification phase.

In the second wave of analysis, the authors utilised the narratives in the data, extant literature, and a process of wānanga (discuss, consider, deliberate and learn) to develop our collective understandings of the literacies that underpinned the words, concepts and metaphors expressed by the participants. The findings presented here represent our identification of examples of literacies and literacy learning, and our analysis of the connection between participants’ narratives and the Mana Model. Within each, we considered the ways that literacies provided the vehicles for learning, and how they might be observed.

## Findings

In this section we discuss five key findings in relation to the types of whānau based learning experiences that affirm the mana of tamariki Māori, and the ‘play’ experiences that facilitate literacy development. Each finding is presented below, along with the voices of participants themselves, which supports the definition of literacies from a te ao Māori perspective. Individual participants remain anonymous in this article, but the wider group from which the narrative derives are defined.

1. Literacies act as the vehicle by which children engage with rich cultural knowledge, tikanga, and as a tool through which they can express every day and abstract cultural meanings.

Māori whānau and kaumātua have known for a long time the importance of identity and belonging, and they are also conduits of cultural literacies that carry this knowledge. The importance of whakapapa (genealogy) and grounding in (often specialised) cultural knowledge was a reoccurring theme in the data. Whānau and kaumātua stated:

*‘My child has learnt her whakapapa/genealogy and pepeha/genealogical belonging. As Māori it is very important, we know who we are and where we come from. We gain a sense of belonging that allows us to be proud of who we are and for this I will always be grateful! My child understands general protocols within our culture, and she demonstrates these wherever she goes. These things along with morals, values…she continues to learn will become fundamental contributions to the strong Māori leader she is becoming.’* (Whānau voice).

*‘[the ideal learning conditions for mokopuna is that they be] … grounded in our cultural values in terms of whakapapa and… they know who they are as Māori and where they fit in this country and in the world.’* (Kaumātua voice).

One whānau member also highlighted that understanding Māori traditions and practices can contribute to a deeper understanding of Māori knowledge retention and identity:

*‘The fact that we have strong tūpuna links, kōrero tuku iho, papa kainga to keep our tamaiti grounded. Knowing who he is, is the foundation of him being able to overcome challenges… He knows his whenua [and his] marae…he walks in it, does mahi on his whenua. [This] [b]uilds his identity.’ *(Whānau voice).

Ensuring tamariki understand the traditional and contemporary relevance of ancestral knowledge is critical to the development of their inherent and inherited literacies. An essential aspect of literacies being a vehicle for engaging in rich cultural knowledge means first acknowledging that diverse Māori knowledge bases (mātauranga Māori) are valid, robust and critical for positive identity development. Māori knowledges are varied, extensive and cohere around the central notion of relationality. According to one Tai Tokerau elder, mātauranga Māori extends across all fields of natural and human endeavour, and our literacies and literacy practices empower children to learn in and through these knowledges:

*‘As Māori, we have expertise in health, we have expertise in whakairo, we have expertise in philosophy, we have expertise in…shall I dare say it, science. In other words, we have a perspective on every topic that exists in the [western education] system.’ *(Kaumātua voice).

Literacies that enable both engagement with and expression of cultural knowledge support children to deepen their understandings of themselves and their place in this world. Mātauranga tuku iho (inherited knowledge), which forms and informs rich cultural knowledge and tikanga (socio-political norms that guide behaviours), includes both tangible and abstract knowledges. Abstract knowledge from Māori a perspective is different to western forms of abstract concepts, but equally as valid. A kaumātua from Tai Tokerau spoke to the complexity of reconciling abstract differences across cultures – specifically Māori and Pākehā cultures:

*‘...this is the conflict we have with each other-to us, we hold on to the wairua. They say to us “I can’t see it, I can’t feel it, so it means nothing.” I’m saying the same thing of the system–“I can’t see it, I can’t feel it, it means nothing to me.” And that’s what we have to come to terms with.’* (Kaumātua voice).

Children accessing knowledge that means something to them is evident in the voices heard from Tai Tokerau. Enabling children to connect with and use what is familiar to them has the potential to enhance education gains and highlight te ao Māori perspectives. Drawing from resources children already have could include familiar pūrākau (stories), such as those extracted from daily life. The importance of observing, becoming aware, seeing where children are at, what their strengths are and how these could be harnessed is evident in whānau voice from Tai Tokerau:

*“Respect their individuality and recognise their strengths and passions. Connect before you correct.'"* (Whānau voice).

(2) Literacies include a wide range of texts that can be individually, culturally and socially constructed and experienced.

Genuine connection supports children to engage with and understand their learning. This includes their participation in social/cultural experiences and cultural meaning making systems as a basis for literacy engagement. Also highlighted by contributors to this study is the importance of children engaging with a wide range of text types that engender diverse learning. These include valued whānau and cultural knowledge conveyed in many Māori literacies, such as weaving, carving, song, story, and also ritual. These texts are expressed as knowledge-sharing and knowledge-seeking expertise, as evidenced by a kaiako from Tai Tokerau:

*‘Students who stand proud in their Māoritanga, can confidently share their taha [M]āori, pepeha and tupuna…[w]hen they have set goals and aspirations for themselves…[h]ave high expectations of themselves and know where they want to go. [When they are] passion driven nothing is going to stop them. Whānau are involved in their child's journey. Whānau voice, student voice, teacher voice. Asking a lot of questions.’* (Kaiako voice).

The value of relationships with cultural practices and literacies enhances what Māori children bring to their learning spaces. The connections tamariki Māori have with people, places and histories have been discussed above along with the integral role multiple generations play in educating a child. Commonly, grandparents and elders were mentioned as key family members who promoted engagement with cultural literacies. The following whānau member highlighted how connection to ancestors and sacred ancestral lands contribute to the process of inheriting literacies and practices that develop cultural identity:

*‘The fact that we have strong tupuna links, kōrero tuku iho, papa kainga…whenua Taurikura. He knows his whenua, marae…he walks in it does mahi on his whenua. Builds his identity.’* (Whānau voice).

The imposition of colonisation has impacted Māori ways of knowing and being socially, culturally, educationally and politically. The educational shift from learning to read the world around us by utilising multiple texts, to the heavy western reliance on print text has been seen by many Māori, including those who shared their knowledge in this project, who lamented the devaluing of Māori literacy practices by the western system. A kaumātua, in reference to their experience of the often forceful western style of teaching reading and writing, which caused disengagement from the intended learning, stated:

*‘...our tūpuna’s [sic] way of educating mokopuna, it was a lot different to this Pākehā…[way of teaching]…The first lesson you learn [from a Māori perspective] is that’s where the kai is [the mother breast], you start learning from there…and you never stop…[from a Pākehā way of learning] you get to a certain age, let’s say five, and then now you must go to school, and you learn Jack and Jill went up the hill…which doesn’t fit with us.’* (Kaumātua voice).

Connecting to the child and their world is a vital aspect in learning that was highlighted multiple times in these data. Notions of learning through role-playing, or by having a role within whānau and beyond (for example iwi roles, roles at school) were highlighted as important literacy practices. The following narrative of one whānau member draws attention to the importance of whānau and connections to people, place, cultural knowledge and values (from multiple sources) is an important way children learn, and an important form of literacy:

*‘They belong and connect to the land and to their family. Aroha. Their connection to people. The ability to navigate different settings and feel at home within them. That they’re a pretty unique mixture of diverse ethnic origins that are united by an understanding of the shared importance of family and people. That displacement from lands has not displaced cultural connection.’* (Whānau voice).

(3) Literacies enable access to knowledge of, and relationships with place accumulated over long periods of time.

In pre-colonial times, ways in which Māori lived, produced, shared and protected their resources supported a political social structure that valued collective well-being. Many of the values and beliefs attributed to this socio-political structure have continued to be passed down through generations, despite the interruption of colonisation. The cultural knowledge that has been reasonably well-preserved as it has traversed generations is often knowledge related to the social and physical aspects of Māori culture. As a kaumātua voice reiterates below, this is perhaps because of Māori connection to and reliance on environments. It may also be because even under the pressure to conform to western practices, many Māori insisted on continuing to live with, on, and in environments as they had for generations. Practitioners therefore had to maintain traditional literacies, and generate new literacies across multiple environments to thrive:

*‘...we were dependent on our environment for our survival. Our knowledge of it, our understandings of its behaviour, and things like that, that’s where our education came from. Our classrooms were either in the garden, or it was up at the mahinga kai’* (Kaumātua voice).

A whānau member also referenced school as an environment where their child could thrive:

*‘My child is thriving in all areas at the moment, and she is absolutely loving school however if I had to choose one area [that]she likes [the] most, it would be kapa haka and everything Māori.’* (Whānau voice).

Many informants to this project referred specifically to the multiple environments and habitats of Papatūānuku (Earth Mother) in relation to literacies, practices and bodies of mātauranga Māori they have inherited, generated and protected over many years. Place-based literacies in relationship to land and sea are deeply rooted in Māori culture. A reason for this may be that as iwi travelled throughout the Pacific they came into contact with varied resources specific to diverse environments. The ability of iwi to not just survive, but to flourish and generate wealth was dependent on how well they could harness and sustain environmental resources. Māori have always adapted and adjusted, but a constant feature of this adaptability has been the deep connections Māori have to place. In modern times this connection has expanded to include modern environments, such as learning environments including schools. An important feature evident in these data was that tamariki who thrived in modern learning environments were able to recognise and participate in te ao Māori literacies, such as kapa haka. From a Māori perspective, at the heart of place-based literacies is the belief in reciprocity–that all relationships between people and place are reciprocal. We are dependent on place and resources, just as the sustainability of place and resources are dependent on people to act with integrity and responsibility.

(4) Reo matatini–literacies are numerous and multi-faceted.

Being literate allows access to understanding and expression of many different literacies. Being literate also requires the ability to make decisions and select the most appropriate skills and tools needed in diverse contexts. Being confident to navigate within different worlds, spaces and contexts require children be multi-literate. A teacher from Tai Tokerau captures the value, richness and multiplicity of te reo Māori and te ao Māori views for educators:

*‘Learn about the iwi and hapū that you teach amongst. Learn and show you value te reo, find out about Te Tiriti o Waitangi and other historical events, take any [professional development] opportunities to do with te ao Māori…make all visitors welcome, learn about Māori protocol, know your students, know their whānau. Value and learn karakia, waiata. Remember kai and the sharing of it is an important part of Māori culture! Laugh a lot. Sing to your students in te reo. Involve the whānau in all aspects of school. Treat them as an asset to you, your students, your school.’* (Kaiako voice).

The many forms of literacy identified in this quote supports our argument for expanding the somewhat restrictive western views and definitions of literacies prevalent in New Zealand schooling (Hetaraka et al., 2023). This kaiako (above) identifies multiple literacies that appeared repeatedly in these data, including whakapapa, history, politics/politicisation, values and ways of being, and beyond. This kaiako also bravely advocates for all teachers to engage in these literacies, to upskill in them to increase the quality of interacting, valuing, sharing and doing to ensure children are thriving.

(5) Whānau are both role models for emerging literacies and are creators of opportunities to harness he inherent strengths of their tamariki.

Relationships that children have with whānau and others around them provide opportunities to learn. Understanding that children enter this world with mana and endless potential is something Māori grandparents are often able to see clearly. Kepa (2015) argued that children need access to competent, confident, and capable adults as an essential element of sociocultural learning. Kepa (2015) also advocates for the return of kaumātua to teaching and learning for the wellbeing and wellness of mokopuna (grandchildren) and whānau.

Family members engage children in social activities that build literacies based on their own knowledge, experiences and expertise. Many of these are shared with children through modelling by whānau members. Literacies develop for children as they engage with knowledge by participating in shared experiences with loved ones, as shared by this Tai Tokerau whānau member:

*‘His koro (East Coast dialect for grandfather) is a great role model as he is a good person, intelligent, gentle, and considerate. He is a hard worker and leads by example.’* (Whānau voice).

Tai Tokerau whānau identify the power of play as a teaching and learning tool. They highlight the value of play for both learning and enjoyment for Māori children:

*‘Her father is very involved and uses play to encourage learning. It is when she is having fun that she gets the most out of her learning.’* (Whānau voice).

In a conversation about what constitutes good role models within a whānau the following exchange was shared with us:

*‘When I asked my son who is a good role model for him, he easily looked at his father and said “Dad”. He explained, “Dad plays plant zombies with me, he is a teacher, and he is Māori”. I asked him is that good? “Yep” he replied.’ *(Whānau voice).

This seemingly simple response brings to the forefront what is of value to this child at this moment in time. A role model for this child is aware of what interests them (playing, plant zombies), includes the role the person holds outside of the home (teacher), and his cultural positioning (Māori) is also of importance. Here, both play and having/playing a role, contributes to socially experienced literacies and making cultural connections.

In response to a question about growing the strengths of Māori students in schooling a Tai Tokerau kaumātua rendered a very simple solution to the potentially endless challenges that many Māori students face in Aotearoa New Zealand schooling:

*“It’s no magical formula, it’s building the child…” *(Kaumātua voice).

This view unpacks and connects to a Māori perspective of literacies for our children–there is no magical formula, significant adults simply need to support, nurture, develop, contribute to and recognise what children inherently and inherited-ly possess. “Building the child” is a fundamentally simple notion made infinitely complex by the circumstances of inequitable conditions that so many children and their families face in modern Aotearoa. Giving children the opportunity to employ and build their skills as generous, courageous, disciplined, humble, reflective, and tenacious participants and leaders is essential.

## Literacy Practices that Create Conditions for Expressions of Mana

This section outlines the interconnected aspects of the Mana Model as defined by Webber and Macfarlane (2020) in relation to the ways they were expressed in this project. Following this, we present further discussion of the multiple ways in which literacies carry, reflect and express important cultural knowledges and practices.

Mana whānau manifests when children experience community connectedness and a strong sense of belonging to people (and place). Children need to feel like they are significant members of the whānau to experiment, take risks, explore, and develop their inherent skills, interests, and strengths. In relation to this project, mana whānau includes inherent and inherited literacies as well as literacy practices maintained, generated and shared through whānau/community interactions. Seeing the mana of a child, or of any person, requires thoughtful observation, deep, considered listening and an understanding of who they come with and from (for example whānau, wider community connections, ancestors). Through the data sets we identified literacies of engagement–conversations, showing and sharing their life–that enabled children to feel confident of their importance in their world(s). These interactions (conceived of here as literacies) relate to Mana Whānau, where whānau play an important role in the types of literacies (here they are largely interpersonal and participatory literacies) available to children so that children develop an awareness of who they are, where they are, why they are. Within Mana Whānau, whānau and community recognise the potential and significance of Māori children within the whānau and the world, this is subsequently translated to children who then also recognise their own potential and significance in their whānau and world.

Mana Ūkaipō manifests when children understand their place in the world and feel a sense of relatedness to all things, both human and non-human. Ūkaipō is a metaphor in te ao Māori for a source of sustenance, origin, or sense of contentedness (Highfield & Webber, 2021). For many Māori, connectedness to place is critical because it is from this sense of tūrangawaewae (place where one has the right to stand) that the concept of relational responsibility forms and solidifies. From a Māori perspective, all things are seen as related as evidenced by the whakapapa (genealogy) of the universe, which is central to Māori epistemology. As this study progressed, the ways in which Māori epistemology and knowledges of the relationality of place/s, the multiplicity of knowledges of place and the differential ways in which these knowledges are shared and learned, came to be conceived as literacies. Therefore, our connections to place and the sense of security fostered by having a strong foundation from which to stand and ever expand are central to the literacies related to the development of Mana Ūkaipō.

Mana Motuhake manifests in children when they develop a positive sense of identity, self-efficacy, and purpose. Mana Motuhake also relates to a perception of one’s opportunities (unrestricted/noa), restrictions (things that might be tapu/sacred) and responsibilities. Mana Motuhake is a complex notion that simultaneously refers to individual agency and collective responsibility. Children who demonstrate Mana Motuhake believe in their own capacity to learn, comprehend, and create knowledge. They also becoming increasingly aware of the responsibility they have to share their learning, expertise and knowledge with others. In this project Mana Motuhake was expressed through the purposeful use of literacies as the vehicle for learning, comprehending and creating. It was also evident that practicing Mana Motuhake involved the regeneration and reignition of pre-colonial Māori knowledge bases and understandings as tools of literacy.

Mana Tū manifests when children not only understand how to learn, but also how to manipulate and reimagine knowledge according to the context. Children learn to understand and deal with difference and adversity by developing competences including courage, tenacity, self-discipline, humility, self-reflection, and kindness. Through Mana Tū children can use cultural knowledge, skills, and abilities to contribute to, and impact on their world/s. Their grasp of literacies, both traditional and contemporary, are the vehicle for communicating their ideas. When embodying Mana Tū children pair their knowledge and skills with culturally significant values such as generosity of spirit, leadership, and gratitude to amplify their actions or messages, which ensures greater impact or influence.

Finally, Mana Tangatarua manifests when children develop the skills, knowledge, and confidence to navigate dual, and/or multiple worlds with emotional, spiritual and intellectual wellness, cultural confidence, and an inclusive mindset. For many Māori, indigenous and marginalised groups, the ability to navigate, interpret and ‘read’ two or more contexts can be considered a literacy skill, which is difficult to understand from a dominant culture perspective. Only those who are forced daily to move in and out of cultural contexts to be understood and to understand can really explain the literacy skills and expertise needed to do so successfully. We saw illustrations of this as a form of literacy in the data used for this project. Māori children must be confident in who they are, and remain tenacious, curious, and motivated to engage with the world/s around them. Children who experience Mana Tangatarua are open to new ideas and doing things differently because of their security in themselves and their cultural belonging. They are more likely to make decisions with moral courage and integrity and are mindful of the values and needs of others.

## Discussion: Literacies as Power-Laden Vehicles of Cultural Knowledges

As an analytical framework, The Mana Model (Webber & Macfarlane, 2020) provided the context for considering how our view of children as maurea, treasures of the highest order, born of and with mana (Hetaraka et al., 2023) might be understood in terms of the conditions that allow them to thrive. During the analyses and wānanga, the model supported us to consider how children access knowledge to support the development of each aspect of mana. Instead of a singular or restricted notion of literacy, a number of different ways of accessing knowledge emerged. Thus, multiple literacies were identified that positioned children as inherently and inherited-ly literate. In this section, we consider varied definitions, and their potential for developing, expressing or passing on knowledge. Whilst the literacies identified here don’t align well with a singular or dominant notion of literacy, the multiplicity itself, identified as literacies, is congruent with western theories of multiliteracies (New London Group, 1996; Cope & Kalantzis, 2015) that are characterised by multiple forms of representation, and multiple languages; however, the notion of tiritira extends literacy beyond a theory of representation, by illuminating the worldview of what is represented.

Knowledge in Māori communities throughout Aotearoa has survived in various forms, through many generations, despite ruptures caused by colonisation. The cultural milieu of iwi was instilled in children not through formal schooling but through their living and participating in all aspects of their community, over a long period of time (Marsden, 2003; Rangihau, 2011). Overarching all the literacies identified in this article was the desire, expressed by the people of Tai Tokerau, that those working alongside children ‘show’ them that te ao Māori, te reo Māori and mātauranga Māori are valued. All of the literacies named by kaumātua and whānau demonstrate that alternatives to Eurocentric positions exist and continue to be successfully practiced within communities.

In traditional times, while Māori parents were busy providing resources and services to the community, grandparents and elders helped to raise children and, in the process, passed down important cultural knowledge via a range of pedagogies (Glasgow & Rameka, 2016, 2017). For teachers, knowing what this knowledge is supports children in making connections between home and school. Through observation, noticing and discussion with children, those who work alongside children are able to make connections to their interests, their passions, their strengths, their whakapapa (genealogy), tūpuna (ancestors) and whenua (land). As evident in this study, a view of literacies that includes te ao Māori perspectives and ways of sharing those perspectives is vital to understanding cultural connections that the child and their whānau value.

By utilising tiritira as a theory of knowledge and viewing the child as maurea, we noticed that literacies were expressed as simultaneously contextualised and timeless. It was apparent that literacies included the everyday interactions with expressions of knowledge, and that these were learned and explored in informal means through everyday interactions of play, talk, participation and imitation. However, also evident was a view of knowledge both passed down and gathered over time using more stable text structures and language forms. Both these perspectives exist in the notion of tiritiria. Literacies mentioned by whānau that demonstrate valued connections and expressions of important learnings were whakapapa, pepeha (identification of important geographical features, places, ancestors, and tribal groupings), pūrākau (stories), stories of their tūpuna (ancestors), kōrero tuku iho (inherited stories), karakia (incantations) and waiata (songs). In this way, the literacies expressed were not only knowledge of these multiple textual forms, but also the ability to use these forms to access the messages passed through the generations; messages that explain cultural values, and carry cultural worldviews. Considering this variety of texts also presents the need for learning to be open to linguistic diversity. Accessing and using texts from Māori perspectives supports the need to work with both English and te reo Māori kupu (words in the Māori language) and content in order for children to make meaning from the depth of understandings that are expressed.

Another cultural value apparent in the data connected to a deeper recognition of the environment. From a Māori perspective, connections to the environment challenge western notions of living and non-living things (Hoskins & Jones, 2017), and also western notions of ‘text’ as expressions of meanings by living things and non-living things. Without delving into the knowledge that underpins this understanding of the world around us, Tai Tokerau whānau spoke of the importance of children connecting to their environment and the everyday expressions of these ideas, for example through the knowledge of kai. This included learning about mahinga kai (places where food is cultivated and/or harvested), being connected to the knowledge and practices of food production in a variety of environments and learning to look after ourselves and our environment in a relationship together. This provides another literacy for us to consider as we reframe and expand the notion of literacies.

Kaumātua and whānau spoke about learning in ways that recognise difference and different ways of teaching–a Māori way and a Pākehā way. They pointed out that many Māori practices that are not acknowledged or promoted in some schools are important for accessing cultural knowledge. A sequence of demonstration, observation and imitation was mentioned, as was recitation. Metge (1995) has referred to role-playing interactions as “learning as part of living” (p. 14) and summarised interactions between kaumātua and mokopuna as having a “main emphasis…on demonstration, observation and imitation” (p. 23).

Intergenerational relationships are also an important component in affirming a sense of belonging. Tai Tokerau participants referenced the significant role of grandparents in the education of grandchildren. The connection between generations is eloquently captured in the definition Eruera and Ruwhiu (2016) provide for the word mokopuna, that “moko can be translated as tattooing or blueprint and puna means a spring of water, therefore a mokopuna is often referred to *as the reflection or blueprint of its ancestors*” (pg. 1).

Relationships in te ao Māori are interconnected, and many Māori protect the array of complex relationships they have with others as a way of maintaining identities. Another relationship type identified as being valuable were tuakana-teina relationships (mentoring premised on familial connection). Tuakana-teina relationships are often seen in school settings but there is somewhat of a knowledge gap regarding the specific role of tuakana-teina relationships for school aged children in literacies and play in Aotearoa.

Recognising and affirming these connections support a sense of belonging for the child. These values include protocols that are applicable in different formal and informal settings. It also includes being able to listen and learn about the people from whom you came, the land, sea and associated pūrakau (stories) and practices. Māori aesthetic texts such as raranga (weaving) and whakairo (carving) are symbolic representations of culturally significant knowledge and histories that can also affirm identity and belonging.

## Conclusion

Using the theoretical underpinning of tiritiria (Hetaraka et al., 2023), the Mana Model framework was utilised as an analytical tool across three existing Tai Tokerau data sets to reframe literacies and literacy practices. Through thematic analysis and wānanga with data and within the research team, we began to identify some of the literacies and practices alive in Tai Tokerau communities. Tai Tokerau voices centred children as significant members of whānau and communities whose knowledges have a history of being silenced by restrictive definitions of literacy and theories of learning. Through this project Māori children have been repositioned as maurea, emphasising the view that each child is unique. This was evident in the analysis as tūpuna, kaumātua, whānau and kaiako spoke to individuality, strengths and passions. In this context, although the child is represented as an individual single, traditionally, each unique individual supported the strength of collective. The many voices of Tai Tokerau have highlighted the ways that literacies in their many forms can be utilised to affirm and grown the inherent and inherited mana of tamariki Māori.

The people of Tai Tokerau represented in the voices throughout this article have much to teach us about literacies and literacy practices from a Māori perspective. Their narratives reflect the need for literacies in Aotearoa New Zealand schools to be framed in ways that affirm the mana of their knowledge bases and their children. The evidence presented here supports the argument that definitions of literacy are constructed by contexts and people; therefore, literacy and literacy practices are value laden. Literacies and their definitions act as the vehicle(s) for how people notice, gather, hold, share, shape, change and contest knowledge together. We have analysed narratives and ideas from our communities in order to re-frame literacies in ways that privilege the diverse funds of knowledge that tamariki Māori and their whānau use daily to communicate and express rich cultural knowledges.
